# Delayed administration of a matrix metalloproteinase inhibitor limits progressive brain injury after hypoxia-ischemia in the neonatal rat

**DOI:** 10.1186/1742-2094-5-34

**Published:** 2008-08-11

**Authors:** Christopher C Leonardo, Autumn K Eakin, Joanne M Ajmo, Lisa A Collier, Keith R Pennypacker, Alex Y Strongin, Paul E Gottschall

**Affiliations:** 1 Department of Molecular Pharmacology and Physiology, School of Basic Biomedical Sciences, College of Medicine, University of South Florida, Tampa, FL, 33612, USA; 2 University of Arkansas for Medical Sciences, Department of Pharmacology and Toxicology, Little Rock, AR, 72205, USA; 3 Burnham Institute for Medical Research, La Jolla, CA, 92037, USA

## Abstract

**Background:**

Hypoxia-ischemia (H-I) can produce widespread neurodegeneration and deep cerebral white matter injury in the neonate. Resident microglia and invading leukocytes promote lesion progression by releasing reactive oxygen species, proteases and other pro-inflammatory mediators. After injury, expression of the gelatin-degrading matrix metalloproteinases (MMPs), MMP-2 and MMP-9, are thought to result in the proteolysis of extracellular matrix (ECM), activation of cytokines/chemokines, and the loss of vascular integrity. Thus, therapies targeting ECM degradation and progressive neuroinflammation may be beneficial in reducing H-I – induced neuropathy. Minocycline has MMP-inhibitory properties and is both anti-inflammatory and neuroprotective. AG3340 (prinomastat) is an MMP inhibitor with high selectivity for the gelatinases. The purpose of this study was to determine whether these compounds could limit H-I – induced injury when administered at a delayed time point.

**Methods:**

Sprague-Dawley rats were exposed to H-I at postnatal day 7 (P7), consisting of unilateral carotid artery ligation followed by 90 min exposure to 8% O_2_. Minocycline, AG3340, or vehicle were administered once daily for 6 days, beginning 24 hours after insult. Animals were sacrificed at P14 for neurohistological assessments. Immunohistochemistry was performed to determine the degree of reactive astrogliosis and immune cell activation/recruitment. Neural injury was detected using the Fluoro-Jade stain, a marker that identifies degenerating cells.

**Results:**

CD11b and glial fibrillary acidic protein (GFAP) immunopositive cells increased in ipsilateral cortex after treatment with vehicle alone, demonstrating microglia/macrophage recruitment and reactive astrogliosis, respectively. Fluoro-Jade staining was markedly increased throughout the fronto-parietal cortex, striatum and hippocampus. Treatment with minocycline or AG3340 inhibited microglia/macrophage recruitment, attenuated astrogliosis and reduced Fluoro-Jade staining when compared to vehicle alone.

**Conclusion:**

The selective gelatinase inhibitor AG3340 showed equal efficacy in reducing neural injury and dampening neuroinflammation when compared to the anti-inflammatory compound minocycline. Thus, MMP-2 and MMP-9 may be viable therapeutic targets to treat neonatal brain injury.

## Background

Exposure to a hypoxic-ischemic (H-I) insult has distinctive consequences in the developing brain. An immature vasculature and low baseline blood flow render the neonatal brain susceptible to even modest changes in perfusion pressure [1]. The maladaptive neurobiological response can be severe, resulting in deep cerebral white matter injury and substantial neuronal loss [2]. Similar to the human condition, neonatal rodent models recapitulating these injuries show cortical and subcortical infarctions, impaired motor function [3-5] and cognitive deficits [6-8]. Previous studies have linked oxidative stress [9,10] and NMDA receptor activation [11] to white matter injury, while glutamatergic blockade has been shown to reduce H-I-induced infarction [12-14] and white matter damage [15]. Though excitotoxicity and free radical production are key contributors to the neuropathology of these lesions, there is a growing interest in identifying additional therapies to limit the progressive neuroinflammation that accompanies ischemic injury.

Inflammation occurs in response to injury and initiates pathological responses that potentiate neural injury. The release of proteases from activated glia results in proteolytic degradation of basement membrane constituents. This breakdown compromises the blood brain barrier, likely allowing entry of peripheral neutrophils and macrophages into the brain. These cells, along with resident microglia, secrete pro-inflammatory cytokines and chemokines that further enhance microglia/macrophage recruitment to the injured site. Matrix metalloproteinases (MMPs), the most well-studied extracellular matrix (ECM)-degrading proteases, are capable of processing TNF-α [16], IL-1β [17] and SDF-1α [18] to their biologically active forms. Several MMPs, particularly the gelatinases, are elevated after cerebral ischemia and have been shown to degrade basement membrane proteins [19,20]. In culture, MMP-2 – positive astrocytes produced MMP-9 when stimulated with either TNF-α or IL-1β [21]. *In vivo*, MMP-2 expression increased in astrocytic endfeet of rats exposed to MCAO, while MMP-9 expression was localized to neutrophils and endothelial cells [22]. Interestingly, elevated gelatinolytic activity colocalized with neuronal laminin degradation after focal ischemia, effects that were attenuated after administration of a highly selective MMP inhibitor [23]. In agreement with these data, mice lacking MMP-9 showed improved outcomes that were directly related to reduced microglial activation [24], attenuated blood brain barrier degradation [25] and limited white matter damage [26] after H-I.

In addition to cytokines and basement membrane proteins, MMPs cleave ECM chondroitin sulfate proteoglycans (CSPGs). Proteolytic processing of ECM proteoglycans has been linked to H-I pathology in the rat neonate [27], and recent data showed altered proteoglycan expression that was associated with progressive injury [28]. While CSPGs are known substrates for several families of matrix-degrading proteases [13,29,30], little is known about the precise regulation of proteoglycan turnover after H-I.

Although neuropathological outcomes show improvement in MMP null mice [24-26], the need for clinically relevant therapeutic intervention remains. In this study, two compounds were selected to determine the effects of MMP inhibition on neuroinflammation and neural injury after H-I. Minocycline, a tetracycline derivative known for its anti-inflammatory properties, is neuroprotective in several rat injury models [31-33] and has recently been shown to inhibit MMP activity both *in vitro *and *in vivo *[34]. AG3340, a small molecule hydroxamate-based inhibitor of MMPs, is efficacious in limiting tumor growth in rodent models [35,36] and was shown to be neuroprotective in adult rodents exposed to chronic ischemia [37]. While both compounds demonstrate good oral bioavailability, AG3340 is a potent MMP inhibitor with high nanomolar affinity for gelatinases, specifically, when compared to the broad anti-inflammatory actions of minocycline. Results here show that both minocycline and AG3340 reduced neuroinflammation and neural injury when administered 24 hrs after H-I, highlighting the potential of targeting MMPs when developing therapies to combat neonatal H-I injury.

## Methods

### Induction of hypoxia-ischemia

All animal procedures were conducted in accordance with the NIH Guide for the Care and Use of Laboratory Animals with a protocol approved by the Institutional Animal Care and Use Committee at the University of South Florida. The number of animals used in this study was limited to the fewest number to complete the project. Neonatal Sprague-Dawley rats were birthed from time-pregnant dams (Harlan Labs). Dams and litters were maintained on a 12 hour light/dark cycle (7 am – 7 pm) and given access to food and water *ad libitum*. Litters from 2 dams were culled to 10 pups per litter at postnatal day 1 (P1) and cross-fostered randomly between dams prior to each experiment. Two H-I experiments consisting of animals from 2 litters per experiment were used in this study. The H-I procedure was originally developed by Levine [38] and reprised by Vannucci and colleagues [39,40] for use in the neonate. The H-I methodology was described in some detail previously [28]. Briefly, the procedure entailed permanent unilateral ligation of the common carotid artery followed by transient exposure to hypoxia. P7 rats were anesthetized with 2.5% isofluorane, placed on a heating pad (37°C) and maintained at 350 ml/min of oxygen and 1.5% isofluorane with an interfaced scavenging system for the duration of the surgery. The right common carotid artery was exposed, isolated away from the vagus and ligated using a 6.0 nylon suture. The musculature and skin were sutured, animals placed back with their corresponding dams for a 2 h recovery period, and subsequently exposed to 8% oxygen/N_2 _balanced for 90 min. During hypoxia, pups were placed into custom-made chambers that maintained a temperature of 37°C while permitting the water-saturated oxygen mixture to be dispensed at a constant flow rate [27]. Pups were then returned to their respective dams until initiation of the treatment phase.

### Drug treatment

Animals exposed to H-I were randomly assigned to receive vehicle, minocycline (Sigma Aldrich, St. Louis, MO) or AG3340 (AG3340 was kindly provided by Dr. Peter Baciu, Allergan, Irvine, CA). Vehicle consisted of 50% DMSO + 25% propylene glycol in distilled water. Minocycline and AG3340 were dissolved in vehicle to obtain stock solutions of 13.5 mg/ml. Fresh stock solutions of drugs were prepared every 2 days. Pups were weighed each day and weight-based injection volumes were calculated to yield a final dose of 45 mg/kg for each animal. Treatments were administered once daily (s.c.) for 6 days beginning 24 hours after H-I (P8). Animals were evaluated daily for signs of pain or discomfort, and no adverse effects were observed in response to either compound or vehicle alone. At the end of the treatment period, animals were sacrificed for histochemical analyses.

### Tissue preparation

For histochemical evaluation, tissues were collected 7 days after H-I (P14) as previously described [28]. Animals were anesthetized with pentobarbital (60 mg/kg) and intracardially perfused with phosphate-buffered saline (PBS, pH 7.4) followed by 4% paraformaldehyde. Brains were removed and cryopreserved with increasing sucrose concentrations (15%, 30%), 30 μm thick sections were cut on a cryostat, and the sections were thaw-mounted onto Superfrost slides (Fisher Scientific, Suwane, GA). Direct mounting onto slides achieved maximum preservation of morphology of the tissue surrounding the lesion. Serial sections were collected throughout the brain beginning at approximately 1.2 mm rostral to bregma and ending at approximately 5.8 mm caudal to bregma.

### Histology and immunohistochemistry

Immunohistochemistry was performed as previously described [28] to assess reactive astrogliosis and microglia/macrophage recruitment to the injured site. Slides were rinsed with PBS, permeabilized and blocked for 60 min (3% Triton-X, 3% 1 M Lysine, 10% NGS in PBS), incubated overnight with primary antibody at 4°C, washed three times with PBS, incubated for 60 min with fluorescent-tagged secondary antibody at room temperature, and coverslipped using Vectashield aqueous mounting media (Vector Labs, Burlingame, CA). Double-label immunohistochemistry was achieved by co-incubation with anti-mouse and anti-rabbit primary antibodies, and subsequent co-incubation with secondary antibodies conjugated to distinct fluorophores for each respective species of primary antibody.

Primary antibodies used in these studies were mouse anti-glial fibrillary acidic protein (GFAP) (Roche Applied Science, Indianapolis, IN; 1:1000) and mouse anti-OX-42 (Serotec, Raleigh, NC; 1:3000). Primary antibodies were visualized using either Alexa Fluor 488 (green) or Alexa Fluor 594 (red) secondary antibodies (Molecular Probes, Eugene, OR). Working concentrations for secondary antibodies were 1:300 for OX-42 and 1:1000 for all other primary antibodies.

In tissue sections from the central nervous system, Fluoro-Jade stain mainly identifies areas of neural injury, predominantly degenerating neurons, and provides a positive quantitative marker as opposed to the absence of signal when using Nissl stain. Fluoro-Jade was previously shown to be a more sensitive measure of neural injury when compared to triphenyltetrazolium chloride [41]. This method was adapted from Schmued and colleagues [42] and subsequently detailed [41]. Tissues mounted on glass slides were sequentially placed in 100% ethanol for 3 min, 70% ethanol for 1 min and deionized water for 1 min. Sections were oxidized for 15 min using 0.06% KMnO_4 _solution followed by 3 brief rinses in PBS. Slides were then immersed in a 0.001% solution of Fluoro-Jade (Histochem, Jefferson, AR) in 0.1% acetic acid for 30 min, rinsed with PBS, dried for 20 min at 45°C, cleared with xylene and coverslipped using DPX medium (Electron Microscopy Sciences, Ft. Washington, PA).

### Image analyses and quantification

Images were acquired using a Zeiss Axioscope 2 (model #801572) controlled by Openlab (Improvision Ltd, Lexington, MA) software, and photomicrographs were captured with a Zeiss Axicam Color (model #412-312) camera. All images subjected to direct comparisons were captured at the same exposure and digital gain settings to eliminate confounds of differential background intensity or false-positive immunoreactivity across sections. Immunoreactivity was quantified using NIH ImageJ software. Photomicrographs of sections from P14 rats were imported into ImageJ and two distinct methods were employed to best assess relative abundance of immunoreactivity. Histogram analyses were performed to assess astrogliosis. Total GFAP intensity values were summed based upon the frequency of positive pixels that occurred within an intensity spectrum ranging from 0 (no immunofluorescence detected) to 256 (highest immunofluorescence intensity). For GFAP quantification, 2 cortical fields per section were selected for analyses. Cortical field selection was achieved by moving lateral from the most dorsal aspect of the corpus callosum (layers 1–4), and again moving medial-ventral to the adjacent field (layers 5–6). For neural injury indicated by Fluoro-Jade stain, the entire cortex was traced and background subtraction was achieved by enhancing contrast until background particles were eliminated from the images. All sections were selected at fixed intervals throughout the bregma coordinates indicated in 'tissue preparation'.

### Statistical analyses

Data from all treatment groups were expressed as X ± SEM. For reactive astrogliosis and cell death, data were represented as total immunofluorescence intensity. Group means were then subjected to a one-way ANOVA with "p value" set at 0.05. Pair-wise comparisons of group means were made using a Dunnett's Multiple Comparison test.

## Results

### Microglia/macrophage recruitment is associated with neural injury

**Figure 1:**
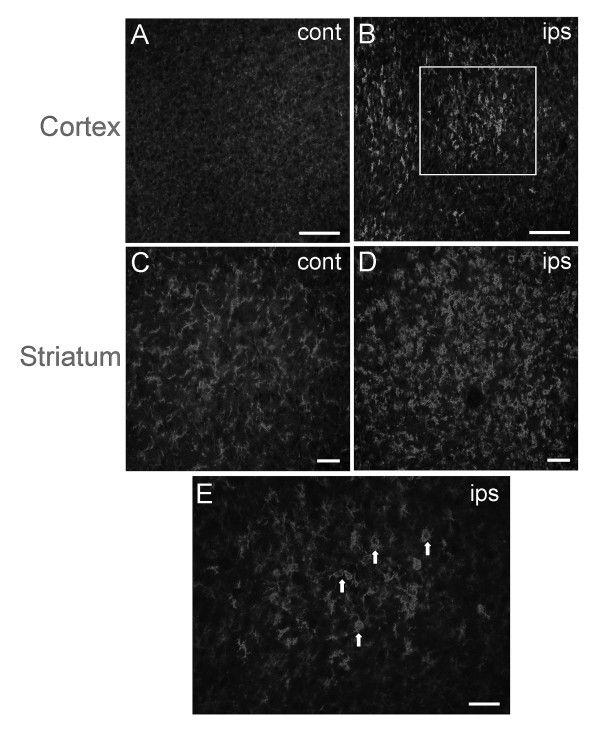
**Microglia/macrophages are activated and recruited to the lesion site 7 days after H-I**. Exposure to H-I at P7 resulted in increased CD11b – positive cells, as measured by OX-42 immunoreactivity, in ipsilateral somatosensory cortex (B) compared to contralateral control (A). CD11b – expressing cells were abundant in corpus striatum of both hemispheres but showed distinct morphology (C,D). Cells in the ipsilateral striatum displayed the activated amoeboid phenotype (D) similar to those in ipsilateral cortex (E) when compared to the ramified morphology in the contralateral hemisphere (C) that is associated with a less activated state. Scale bars = 100 μm (A,B), 50 μm (C-E).

Exposure of the neonate to H-I initiates injurious biochemical cascades that facilitate recruitment of pro-inflammatory cells to the lesion site. An early and sustained indicator of CNS injury is the activation and recruitment of microglia and macrophages to the injured site. These cell types are important in the initiation and maintenance of the neuroinflammatory response, including the production of MMPs. Immunohistochemistry was performed on sections from untreated and vehicle-treated animals that were exposed to H-I (N = 5 per group) to assess the immune cell response using anti-OX-42, an antibody that binds the CD11b antigen that is expressed on cell surfaces of microglia and macrophages. Intense OX-42 immunoreactivity was detected 7 days after H-I (Figure 1). Robust elevations in OX-42 – positive cells occurred in the ipsilateral cerebral cortex (Figure 1B) when compared to the contralateral control region (Figure 1A). Although some labeling was detected in the contralateral cortex, immunoreactivity was faint and diffuse by comparison. In contrast, cells expressing the CD11b antigen were abundant throughout the striatum of both hemispheres (Figure 1C,D). Closer examination revealed that OX-42 – positive cells displayed an amoeboid morphology in the ipsilateral striatum (Figure 1D) and cortex (Figure 1E) that is consistent with activated microglia or macrophages, while CD11b – expressing cells in contralateral striatum exhibited a ramified morphology.

**Figure 2:**
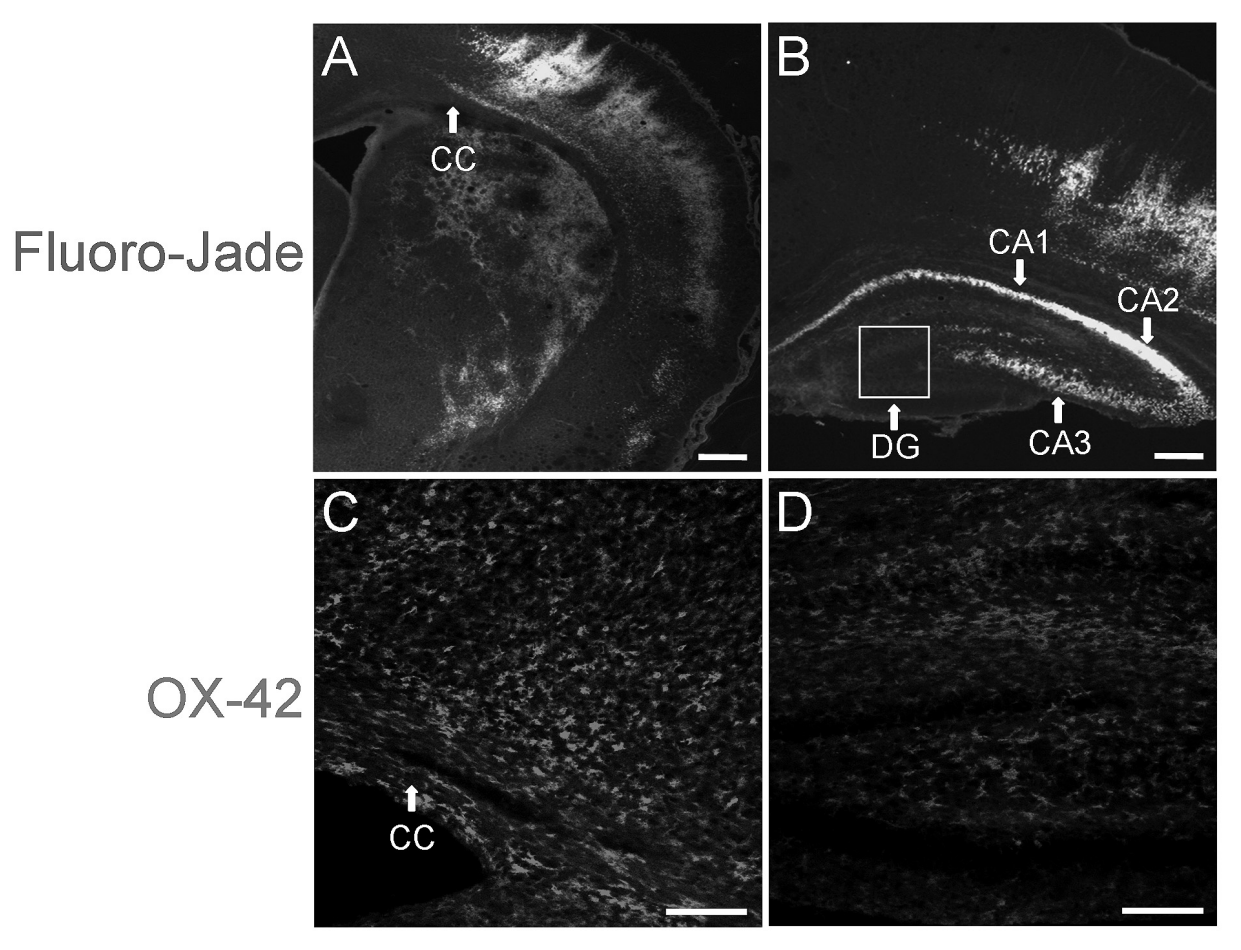
**Microglia/macrophage activation and recruitment are associated with neural injury**. Exposure to H-I at P7 resulted in robust Fluoro-Jade staining throughout cerebral cortex, corpus striatum (A) and CA1–CA3 pyramidal cell layers of hippocampus (B), indicating severe neural injury. OX-42 immunoreactivity showed CD11b – expressing cells at the cortical lesion site (C). Hippocampal OX-42 localized to the molecular layer of dentate gyrus and was evident in hippocampal regions seated ventral to CA1, but was not present in the pyramidal cell layers (D). Scale bars = 100 μm.

Hypoxia-ischemia often produces cortical and subcortical cavitary infarctions over time that are not present up to 4 days after insult. Tissues from vehicle-treated animals (N = 5) were collected 7 days after H-I and stained with Fluoro-Jade to determine the degree of neural injury present at this time (Figure 2). Fluoro-Jade staining was prominent in the ipsilateral hemisphere. Signal appeared as columns in lower cortical layers and was diffusely distributed throughout the striatum (Figure 2A). The cortical and striatal injury profile was consistent with elevated OX-42 immunoreactivity (Figure 2C, Figure 1D). Fluoro-Jade also stained hippocampal CA1–CA3 pyramidal neurons intensely (Figure 2B), while other hippocampal fields showed little staining. OX-42 immunoreactivity did not colocalize with Fluoro-Jade in the pyramidal cell layers. However, CD11b – positive cells were localized to the molecular layer of dentate gyrus and other regions seated ventral to CA1 (Figure 2D). In general, Fluoro-Jade staining was present in and adjacent to regions that contained increased numbers of activated microglia/macrophages, indicating that the activation of these cells is associated with neural injury.

### Delayed treatment dampens reactive astrogliosis

**Figure 3:**
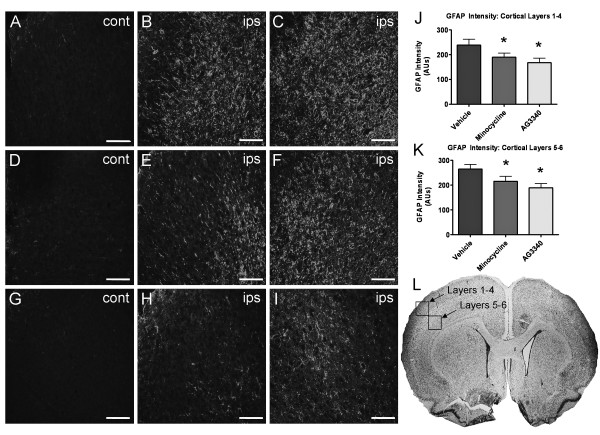
**Delayed treatment with minocycline or AG3340 dampens reactive astrogliosis**. P7 rat pups were exposed to H-I and treated with either vehicle, minocycline or AG3340 once daily for 6 days, beginning 24 hours after insult. (A-C) Astrogliosis, as measured by GFAP immunoreactivity, was markedly elevated in cortical layers 1–4 (B) and layers 5–6 (C) compared to contralateral control (A) after treatment with vehicle alone. Administration of either minocycline (D-F) or AG3340 (G-I) 24 hours after H-I reduced GFAP immunoreactivity in layers 1–4 (E,H) and layers 5–6 (F,I) compared to control hemisphere (D,G). GFAP immunoreactivity was significantly reduced in cortical layers 1–4 (J) and layers 5–6 (K) of animals treated with either minocycline or AG3340 compared to treatment with vehicle alone. N = 8, * = p < 0.05. Scale bars = 100 μm.

Previous data demonstrated a robust astroglial response early after H-I in the neonate, indicating that reactive astrocytes are key players in neuroinflammation and respond upstream of severe infarction [28]. To test whether inhibition of MMPs can reduce astrocyte reactivity after H-I, immunohistochemistry was performed 7 days after H-I to assess GFAP upregulation (Figure 3) in animals treated with vehicle, minocycline or AG3340 for 6 days after H-I. Low levels of GFAP were detected in contralateral cerebral cortex (Figure 3A,G). In general, GFAP immunoreactivity was prominent in ipsilateral somatosensory cortical layers 5–6 (Figure 3C,F,I) with lesser relative intensity in cortical layers 1–4 (Figure 3B,E,H). Animals treated with vehicle alone showed robust increases in GFAP in layers 1–4 (Figure 3B) and layers 5–6 (Figure 3C). This is consistent with the astroglial response previously reported at an earlier endpoint [28]. Astrogliosis was diminished after administration of either minocycline or AG3340. While the observed effect of minocycline was most evident in cortical layers 1–4 (Figure 3E), administration of AG3340 diminished astrogliosis in layers 1–4 (Figure 3H) and appeared more efficacious in layers 5–6 (Figure 3I) when compared to minocycline (Figure 3F). Quantification was performed by analyzing photomicrographs (N = 8 per group, 5 sections per animal) for total GFAP immunoreactivity. Results showed significant reductions in GFAP immunoreactivity in cortical layers 1–4 (Figure 3J) and layers 5–6 (Figure 3K) of animals treated with either minocycline or AG3340 compared to treatment with vehicle alone (p < 0.05). Despite a clear trend toward diminished fluorescent signal in sections from animals treated with AG3340 relative to minocycline, efficacy did not differ significantly between treatments.

### Delayed treatment reduces neural injury

**Figure 4:**
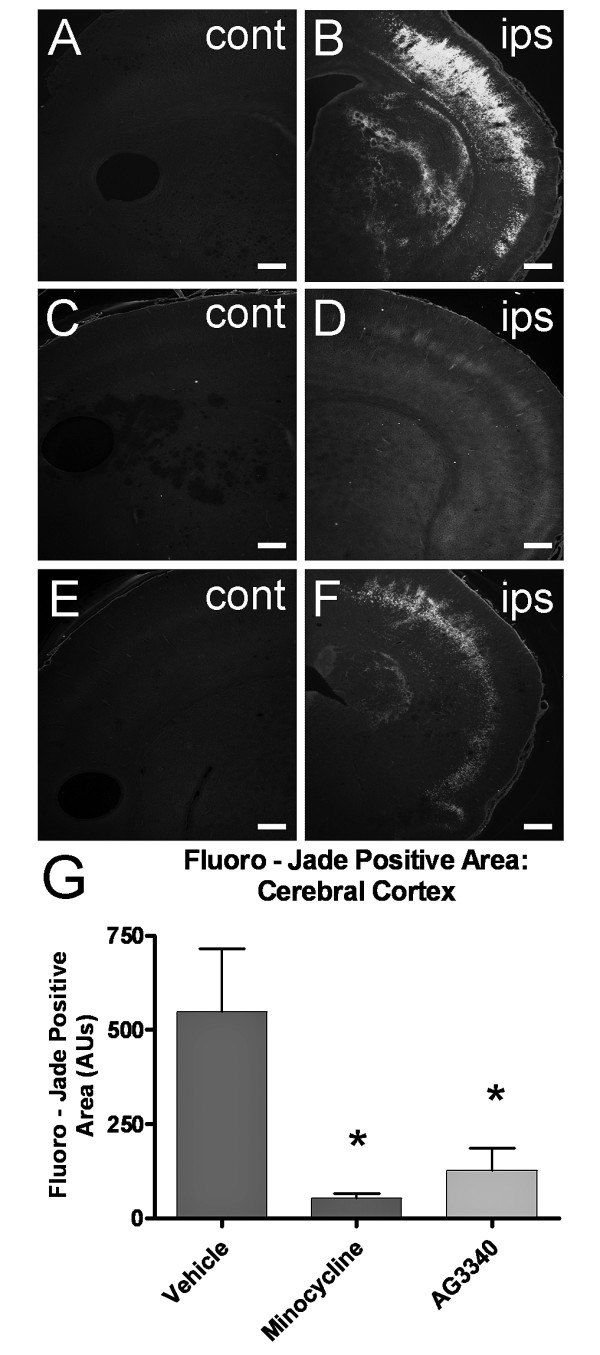
**Delayed treatment with minocycline or AG3340 reduces neural injury**. P7 rat pups were exposed to H-I and treated with either vehicle, minocycline or AG3340 once daily for 6 days, beginning 24 hours after insult. Animals treated with vehicle alone showed robust Fluoro-Jade labeling in ipsilateral cortex and corpus striatum (B). Fluoro-Jade staining was nearly abolished after treatment with minocycline (D) and greatly reduced after treatment with AG3340 (F) relative to vehicle alone (B). No labeling was detected in contralateral control regions (A,C,E). Quantification showed a significant reduction after treatment with either minocycline or AG3340 (G) compared to treatment with vehicle alone. N = 5, * = p < 0.05. Scale bars = 100 μm.

Marked increases in Fluoro-Jade staining were evident in ipsilateral cerebral cortex, corpus striatum and hippocampus of animals exposed to H-I and treated with vehicle alone (Figure 2, 4). Staining was absent from the contralateral hemisphere and very little non-specific background staining was detected (Figure 4A,C,E). Intense columnar-shaped immunofluorescence was evident throughout the cerebral cortex in sections that ranged from the initial emergence of lateral ventricles to ventral hippocampus. Treatment with minocycline abolished this effect, showing little or no cortical Fluoro-Jade labeling (Figure 4D). Administration of AG3340 also reduced Fluoro-Jade staining in cerebral cortex (Figure 4F) when compared to sections from animals treated with vehicle alone, though this compound appeared to be less efficacious than minocycline. Quantification of cortical Fluoro-Jade staining was performed by analyzing photomicrographs (N = 5 per group, 5 sections per animal) for total area occupied by stain. Results showed a significant reduction in Fluoro-Jade staining after treatment with both compounds (Figure 4G) when compared to treatment with vehicle alone (p < 0.05). Efficacy did not differ significantly between minocycline and AG3340.

### Delayed treatment ameliorates microglia/macrophage response

**Figure 5:**
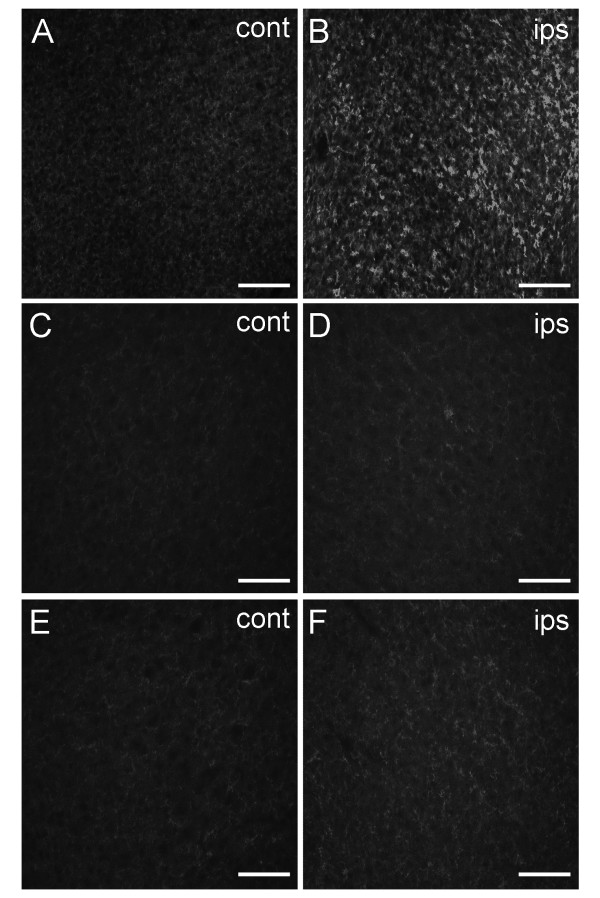
**Delayed treatment with minocycline or AG3340 ameliorates the microglia/macrophage response**. P7 rat pups were exposed to H-I and treated with either vehicle, minocycline or AG3340 once daily for 6 days, beginning 24 hours after insult. Intense OX-42 immunoreactivity was evident throughout the ipsilateral cerebral cortex of animals treated with vehicle alone (B). Microglia/macrophage labeling was nearly abolished after treatment with minocycline (D) and was markedly reduced after treatment with AG3340 (F). OX-42 immunoreactivity in contralateral control regions was faint and diffuse by comparison (A,C,E). Scale bars = 100 μm.

To determine whether reduced cell death was associated with microglia/macrophage recruitment to the lesion site, immunohistochemistry was performed with anti-OX-42 to detect the CD11b antigen present on cell membranes of microglia and macrophages. Tissues from animals treated with vehicle alone showed increased OX-42 immunoreactivity in the ipsilateral cerebral cortex (Figure 5B). CD11b labeling in contralateral control regions was faint and diffuse by comparison (Figure 5A), indicating that OX-42 – positive cells were recruited to the injured area or that those present markedly increased the expression of CD11b. Treatment with minocycline ameliorated this effect, as ipsilateral OX-42 immunoreactivity (Figure 5D) was indistinguishable from that observed in the contralateral control hemisphere (Figure 5C). OX-42 – positive cells were also greatly reduced in ipsilateral cortex of animals treated with AG3340 (Figure 5F) relative to contralateral control (Figure 5E).

## Discussion

The central nervous system response to hypoxia-ischemia or ischemia reperfusion injury often includes activation of astrocytes, resident microglia, and the infiltration of peripheral leukocytes. Subsequent release of pro-inflammatory cytokines and other inflammatory effectors contribute to neural injury of white and gray matter after H-I [43]. The present study evaluated the degree of reactive astrogliosis, microglia/macrophage recruitment and neural injury after H-I in the P7 Sprague-Dawley rat neonate. A single daily administration of minocycline or AG3340 for 6 days, beginning 24 hrs after H-I, provided protection against neural injury and dampened astrocyte and microglial activation induced by the insult. Because of the progressive nature of the insult that follows H-I, it is likely that inhibition of inflammatory effectors by minocycline or AG3340 was associated with reduced neural injury.

The neurobiological reaction to H-I-induced lesion involves a progressive inflammatory response that exacerbates the injury via the release of cytokines, chemokines and other effector molecules from reactive astrocytes, resident immune cells and peripheral leukocytes. Robust elevations in GFAP immunoreactivity were prominent throughout the ipsilateral cerebral cortex of animals treated with vehicle alone. Fluoro-Jade histochemistry revealed marked neural injury that was anatomically consistent with heightened GFAP and OX-42 labeling. CD11b – positive cells were abundantly distributed throughout the cortex, striatum and hippocampus after insult. Evidence indicates that resident microglia, and not infiltrating leukocytes, are the primary immune cells that respond to ischemic injury in the neonatal rat brain [44]. Although the majority of CD11b – expressing cells detected here displayed an amoeboid morphology characteristic of activated microglia, it is also possible that these cells were peripheral monocytes that had entered the brain after insult.

MMPs, derived mainly from microglia, influence the neurological outcome after hypoxia and/or ischemia-induced lesion in a complex manner. After experimental stroke, MMP-9 null mice showed reduced blood brain barrier degradation and white matter injury [26]. However, more recent data demonstrated that acute expression of MMPs contributes to injury, yet more chronically, MMPs promote plasticity and recovery [45]. Most relevant, the extent of neural injury induced by H-I in the rat neonate was diminished in MMP-9 null mice compared to the lesion in wild type mice [24]. MMP-9 co-localizes with activated resident microglia [24] and infiltrating leukocytes [25,46] in rodents subjected to cerebral ischemia, indicating a link between activated immune cells and MMP expression. Consistent with these data, the absence of immune cell recruitment after treatment with inhibitors in the present study provides further evidence that MMP activity is linked to neuroinflammation.

Several different mechanisms could account for the neuroprotection afforded by AG3340. MMPs participate in complex injury responses through interactions with and activation of cytokines, chemokines and other pericellular and cell surface substrates [47], and inhibition would limit the action of these pro-inflammatory agents [16,45]. Because gelatinases efficiently degrade basement membrane proteins, it is possible that the actions of the MMP inhibitor resulted in preservation of basement membrane proteins and an intact blood brain barrier. Another possible mechanism of the MMP inhibitor could be that a loss of ECM proteolysis derived from microglia limited their ability to migrate through the ECM to the region of injury (or other unknown mechanisms). This is an interesting potential mechanism which, to date, has not been investigated. Data from the present study indirectly support such a mechanism since microglia/macrophage density in the injured cerebral cortex was diminished in animals treated with AG3340. Additionally, results here showed that delayed administration of AG3340 24 h after H-I limited microglial numbers and conferred protection from neural injury. This suggests that selective targeting of MMPs even after an episode of H-I may be a clinically relevant therapeutic approach.

Minocycline is known for its broad anti-inflammatory actions and has recently been shown to inhibit MMP activity in an *in vivo *stroke model [34]. In the present study, we sought to determine the degree to which two compounds that exhibit distinct anti-inflammatory actions and affinities for MMPs, differentially influence neural injury caused by H-I in the neonate. In a manner similar to AG3340, minocycline reduced microglia/macrophage recruitment and cortical injury after H-I. Both compounds also inhibited astroglial reactivity in the injured region, suggesting that MMPs may contribute to the activation of astrocytes as well. Two other neonatal H-I studies have demonstrated short-term [31] and long-term [32] protection after treatment with minocycline. Importantly, these experiments differed in that animals were pretreated prior to insult and again immediately after occlusion, whereas in the present study, treatment did not begin until 24 hours after the insult.

## Conclusion

The present study indicates that treatment with either AG3340, a compound that is relatively selective for gelatin-degrading MMPs, or minocycline, a tetracycline derivative with anti-inflammatory and MMP-inhibitory properties, protected the neonatal brain even when first administered 24 h after H-I, a clinically relevant time point. Future investigations are necessary to define the early time course for efficacy, the specific pro-inflammatory mechanisms involved in progressive H-I injury, and the degree to which the neuroprotective effects of these compounds are dependent on MMP activity. These studies, in conjunction with data reported here, will aid not only in identifying the specific sequence of neuroinflammatory events that contribute to H-I injury, but also in determining whether MMPs mediate these processes and therefore represent attractive therapeutic targets.

## Competing interests

The authors declare that they have no competing interests.

## Authors' contributions

CCL and PEG designed the experiments. CCL performed the H-I procedure, drug injections, brain sectioning, immunohistochemistry, data analysis, and wrote the manuscript. AKE assisted with sectioning and immunohistochemistry. LAC performed the Fluoro-Jade histochemistry. PEG directed this work, contributing to the data analysis and writing of the manuscript. All authors edited and approved the final manuscript.
